# Improved production of monoclonal antibodies against the LcrV antigen of *Yersinia pestis* using FACS-aided hybridoma selection

**DOI:** 10.14440/jbm.2018.257

**Published:** 2018-11-07

**Authors:** Assa Sittner, Adva Mechaly, Einat Vitner, Moshe Aftalion, Yinon Levy, Haim Levy, Emanuelle Mamroud, Morly Fisher

**Affiliations:** 1 Department of Infectious Diseases, Israel Institute for Biological Research, P.O. Box 19, Ness Ziona 74100, Israel; 2 Department of Biochemistry and Molecular Genetics, Israel Institute for Biological Research, P.O. Box 19, Ness Ziona 74100, Israel

**Keywords:** FACS, hybridoma, sorting, selection

## Abstract

For about four decades, hybridoma technologies have been the “work horse” of monoclonal antibody production. These techniques proved to be robust and reliable, albeit laborious. Over the years, several major improvements have been introduced into the field, but yet, antibody production still requires many hours of labor and considerable resources. In this work, we present a leap forward in the advancement of hybridoma-based monoclonal antibody production, which saves labor and time and increases yield, by combining hybridoma technology, fluorescent particles and fluorescence-activated cell sorting (FACS). By taking advantage of the hybridomas’ cell-surface associated antibodies, we can differentiate between antigen-specific and non-specific cells, based on their ability to bind the particles. The speed and efficiency of antibody discovery, and subsequent cell cloning, are of high importance in the field of infectious diseases. Therefore, as a model system, we chose the protein LcrV, a major virulence factor of the plague pathogen *Yersinia* pestis, an important re-emerging pathogen and a possible bioterror agent.

## INTRODUCTION

Antibody production is a field of utmost importance, especially in the context of detection and treatment of select agents and emerging diseases. Despite many developments in the field, hybridoma monoclonal antibody production still shows unmatched robustness and reliability [1,2]. However, the labor required to produce a large collection of antibodies using this method grows exponentially with the number of clones. Thus, as antibody characterization methods move into the high-throughput era [3], hybridoma antibody production routines still remain the rate limiting step in antibody development. In order to address these issues and increase the yield of traditional hybridoma techniques, we chose to take advantage of fluorescence-activated cell sorting (FACS). While traditional hybridoma techniques use “blind” distribution of hybridomas into microplates, followed by a process of limiting dilution of positive wells, FACS methods sort the contents of the fusion and select only hybridomas that bind to a fluorescently labeled antigen. Moreover, recent sorter systems allow efficient sorting of single cell events, thereby making the cloning step unnecessary [4]. In the work presented here, we conjugated fluorescent particles with the antigen, and let them bind to hybridomas that present the specific antibody on their surface. This way, only “antigen-specific” hybridomas bind the particles and become fluorescently labeled, allowing cytometric sorting. The clones are then tested by an enzyme-linked immunosorbent assay (ELISA), to characterize the binding properties of the antibodies. Only recently, a similar work has been published, which used soluble, fluorescent molecules [5]. This approach relies on strong antibody display on the cell surface, as the fluorescent signal is proportional to the number of labeled antigen molecules that bind to it. In the method we present here, the binding of large fluorescent particles, rather than soluble proteins, to the hybridomas can produce a strong and discrete fluorescent signal even in cases where the surface display of antibodies is relatively sparse. Together, the two mutually complementary methods demonstrate the great promise that FACS holds for antibody development. We chose to implement our methodology on *Yersinia* (*Y*.) *pestis*, the causative agent of plague, both a potential bio-terror [6] agent and a re-emerging pathogen [7]. The virulence of *Y. pestis* relies on the type III secretion system (T3SS), which injects proteins into host cells. LcrV, which plays a key role in pathogenesis, is an important component of the T3SS injection body and a potent protective antigen against plague. It has been shown that passive immunization by transfer of anti-LcrV antibodies represents a valuable post-exposure therapeutic approach, able to protect animals against both bubonic and pneumonic plague [8-10]. The localization of LcrV to the tip of the T3SS needle [11], suggests that the observed protective effect elicited by administration of antibodies may result from prevention of the translocation of effectors through the injectisome. Therefore, the development of antibodies against LcrV is motivated by two major reasons: Firstly, the emergence of antibiotic-resistant *Y*. *pestis* strains [12] stresses the importance of non-antibiotic treatments, for which anti-LcrV antibodies may serve as an attractive option. Secondly, anti-LcrV antibodies can be used for the diagnosis of plague infection by detecting soluble LcrV in patient blood [13].

## MATERIALS AND METHODS

### Ethical statement

This study was performed in accordance with the recommendations for the Care and Use of Laboratory Animals (National Institutes of Health [NIH]). Animal experiments were performed in accordance with Israeli law and were approved by the Institutional Ethics Committee for animal experiments at the Israel Institute for Biological Research (Permit number: IACUC-IIBR M-08-2015).

### Preparation of recombinant *Y. pestis* LcrV

The full-length lcrV coding sequence from the fully virulent Y. pestis strain Kimberley53 was cloned into the pGEX expression vector (GE Healthcare) and introduced into MC1060 *E. coli* cells. Recombinant LcrV was produced and purified as described previously [14]. Endotoxins were removed from the purified protein preparations by the Triton X-114 phase-separation method [15]. The endotoxin levels that were measured by the LAL method were < 1 IEU (international endotoxin units)/50 μg of the purified LcrV protein.

### Vaccination of mice with rLcrV

For prime vaccination, recombinant LcrV antigen (50 g) was mixed with complete Freund’s adjuvant (Sigma, Israel) and for boost vaccinations, a similar dose of the antigen was mixed with incomplete Freund’s adjuvant (Sigma, Israel). BALB/c mice (Charles River) received three intraperitoneal injections of the adjuvant-antigen mixture (100 L/dose) separated three weeks apart. Blood samples were taken prior to each vaccination and anti-LcrV IgG titers were determined by ELISA as previously described [9]. Three days of the last immunization, mice were sacrificed and splenocytes were collected from the spleen in PBS. The cells were counted and fused with NS0 cells at a ratio of 1:10 using freshly prepared 50% polyethylene glycol MW 1500 (Sigma, Aldrich) [16]. After extensively washing the fusion mixture with RPMI 1640, the cells were seeded in RPMI supplemented with 10% fetal Bovine Serum and HAT [17] (Biological Industries, Israel) in ten 90 mm culture dishes for six days.

### Cell cultures

Mouse NS0 cells [18] (ECACC No. 85110503) were cultured in RPMI 1640 (Biological Industries, Israel) with 10% heat–inactivated fetal bovine serum (Biological Industries, Israel).

### Preparation of LcrV-conjugated fluorescent particles

Conjugation of LcrV to amine-coated, fluorescent Nile blue particles (Spherotech) was performed using EDC and Sulfo-NHS (ThermoFisher) by a two-step procedure [19,20]. Briefly, purified LcrV at a concentration of 1 mg/ml was activated with EDC and sulfo NHS (Thermo) in MES buffer, pH = 6.0 for 30. After quenching excess EDC with β-mercaptoethanol (Sigma), the fluorescent particles were added in carbonate buffer, pH = 8.3. The LcrV conjugated particles were then washed three times in PBS and stored at 4°C.

### Validation of particle binding to hybridomas

10^6^ hybridomas from a reference, anti-LcrV line, were washed twice with RPMI and incubated for 30 in a rotator at room temperature with either LcrV-or BSA-conjugated particles. Then, the cells were washed twice with RPMI and placed on coverslips coated with poly-L-lysine (Sigma). The cells were then stained with DAPI (Sigma) and mounted with fluoromount on glass slides (Sigma). Imaging was performed using a confocal microscope (Zeiss LSM 710).

### Sorting

For the sorting, 10% of the fusion plate were washed twice with RPMI, mixed with LcrV-coated particles, and incubated for 30' with mild shaking. Then, the tube was taken to the FACS (BD FacsAria III), for a brief analysis run. The run was used to determine the gating parameters for positive clones. Immediately following the analytical run, we initiated a sorting run, which sorted the contents of the fusion plate into a 96 well plate in a single cell mode, at room temperature. In order to obtain an effectively monoclonal sorting, we sorted 10 cells per well, which according to our analysis, should produce single clones with high probability (see Cloning). In order to verify monoclonality, the hybridoma colony formation was followed on a daily basis using an inverted microscope (Nikon Eclipse Ts2). The analysis showed the development of single viable cells to single colonies per well, which later developed to large colonies For extra safety, we performed another subcloning step, using limiting dilution.

### Hybridoma supernatant processing and biolayer interferometry

Each hybridoma, positively sorted by the FCM sorter, was grown RPMI medium supplemented with 10% NRS, Antibiotic, Thymus extract (2.5% H6020-sigma). The supernatants from the ELISA-positive clones and a non-related clone (negative control) were collected and concentrated using an Amicon centrifugal filter device with a MW cutoff of 30 K (Merck Millipore). The concentrated supernatants were washed once with Octet buffer (PBS buffer, pH 7.4, containing 10 mg/ml BSA and 0.1% (v/v) Tween 20) to a final volume of 1 ml. Antibody binding studies were carried out using the Octet Red system (Forte Bio). All steps were performed at 30°C with shaking at 1500 rpm in a 96-well plate containing 200 μl solution in each well. Octet buffer was used throughout this study for antibodies and analytes dilution and for washing the sensors. Streptavidin-coated biosensors were loaded with a mouse anti-LcrV biotinylated antibody (5 μg/ml) for 300 s followed by a 60 s wash. The sensors were then exposed to LcrV (5mg/ml) for 300 s, washed again for 60 s and submerged in wells containing the concentrated hybridoma supernatant of the different antibodies for another 300 s followed by another 60 s wash. As a positive control, a polyclonal anti-LcrV antibody was used. A supernatant from a non-related mouse monoclonal hybridoma was used as a negative control in the assay.

### Cloning

Due to the high sensitivity of hybridomas, their survival rates in BD FacsAria III’s “single cell” sorting mode were low, and therefore not practical for producing a satisfactory number of viable clones in reasonable time. In order to cope with this problem, we used a higher number of hybridomas per well, as a means to achieve “effective monoclonality”. We first determined the survival rates of hybridomas following sorting in room temperature, using a “survival test” sort for hybridomas. This was done by sorting different numbers of hybridomas per well into 96 well plates and analyzing the survival statistics. These statistics were used to re-calibrate the sorting conditions, in order to obtain single clones with a high probability.

In order to obtain the survival probability for a single cell, we assume a simple binomial survival model, in which the probability for a given sorted cell to survive, *P*_survive_, is constant. In such a model, the probability for *n* viable hybridomas (capable of forming a colony) in a well, given that *N* hybridomas were sorted into the well, is given by the binomial probability mass function 
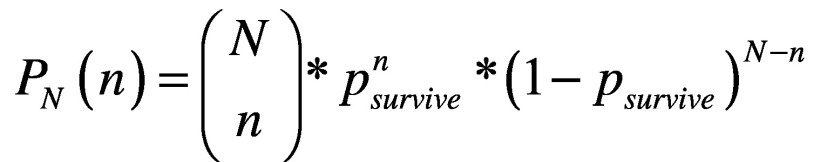
.

The probability for observing growth in a well (or non-zero viable hybridomas in a well) is: 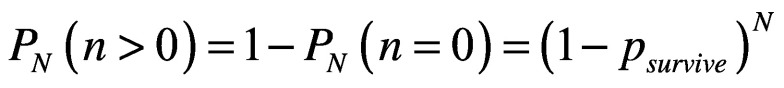.

By counting the wells in which hybridomas grew in the calibration experiment, we found that when *N* = 10, 37.5% of the wells were positive (36/96 wells), and therefore, 
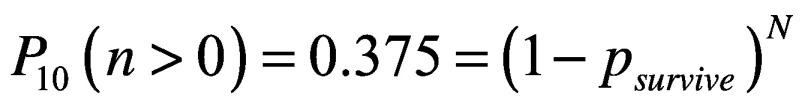
.

In such a case, *P*_survive_ = 0.023 and the probability for a well with no viable clones, using the binomial model, is *P*_10_(*n* = 0) = 0.79, and the probability for a single clone is *P*_10_(*n* = 1) = 0.19, strongly decreasing with *n*. Given that a well shows growth (which can easily be determined within a few days from the sorting), the probability that it would be monoclonal is 
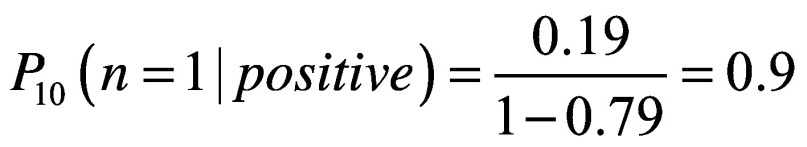
, so in large numbers, 90% of the wells in which hybridomas grow will be monoclonal.

### Data analysis

Analysis and plotting of .fcs files was performed on FlowJo v10. Additional statistical analysis and plotting was performed using the Scipy programming library for python [21].

## RESULTS

### Validation of system components

**Figure 2.**
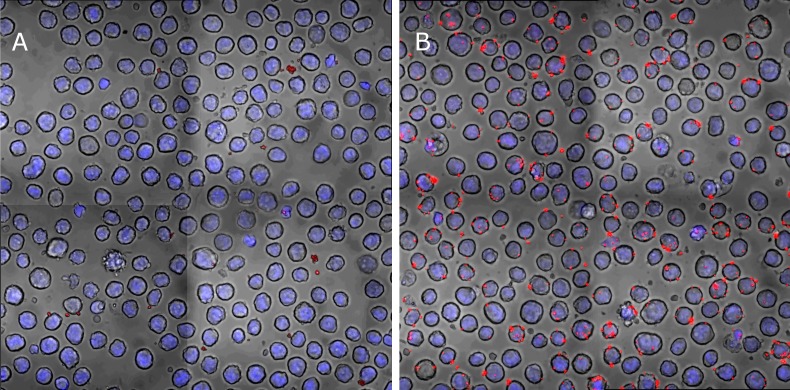
Binding of LcrV-conjugated particles to anti-LcrV hybridoma cells. **A.** Control particles conjugated with BSA do not bind to the hybridomas. **B.** LcrV-conjugated particles bind to anti-LcrV hybridomas but do not form aggregates.

Most of the hybridoma screening methods developed so far, are based on the characterization of antibodies secreted by hybridomas into the medium. The sorting method, however, relies on the display of the antibodies on the outer membrane of the hybridoma, in a way that allows the cell, as a whole, to bind the fluorescently-labeled antigen and therefore to be sorted by FACS. The conjugation of the antigen (LcrV) to the beads was found to be stable for at least three days at 4°C in PBS, which was verified using a qualitative immunoassay. Next, we used microscope analysis to show that the LcrV-conjugated particles bind existing anti-LcrV hybridomas. As can be seen in **Figure. 2**, the hybridoma cells bind the fluorescent particles and not the negative control (particles conjugated with BSA). Moreover, under these conditions, the cells are monodisperse, *i.e.*, the particles do not form “bridges” between cells, which may cause aggregates. This may also be an indication of a low number of surface-conjugated LcrV molecules per particle. Finally, we analyzed the binding cytometrically. The results (see **Fig. 2** in supplementary information) clearly show binding, and provide initial gating parameters for the sorting of freshly-fused hybridomas.

### FACS-aided hybridoma sorting *vs*. the traditional hybridoma method—an efficiency comparison

**Figure 3.**
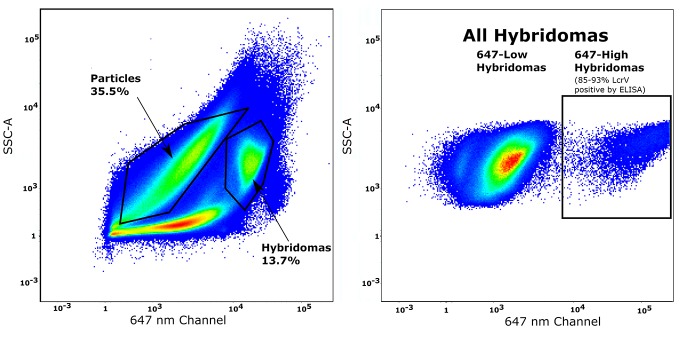
Sorting freshly-fused hybridomas using the fluorescent signal of LcrV-coated particles. Left: Hybridomas were selected using light scattering properties. Right: Hybridomas from the light scattering gate were analyzed according to the intensity in the 647nm channel (Nile blue) and then sorted.

Spleens from LcrV-vaccinated mice were harvested for fusion with myelomas, after which the resulting cells were divided into two unequal groups. Ten percent of the cells resulting from the fusion experiment were used in sorting. As a control experiment, we processed the other 90% of the cells from one of the spleens, using the traditional limiting dilution protocol. One week post-fusion, the hybridomas were incubated with the Nile blue labeled, LcrV-conjugated particles and then sorted by FACS. About 40000 cells were gated using basic light scattering properties and analyzed for their fluorescent signal using a 647 nm filter set, to detect the Nile blue fluorescence of associated particles. The hybridomas that were bound to the Nile blue beads appeared as a well-resolved subpopulation (**Fig. 3**), which was sorted into a 96-well plate.

**Figure 4.**
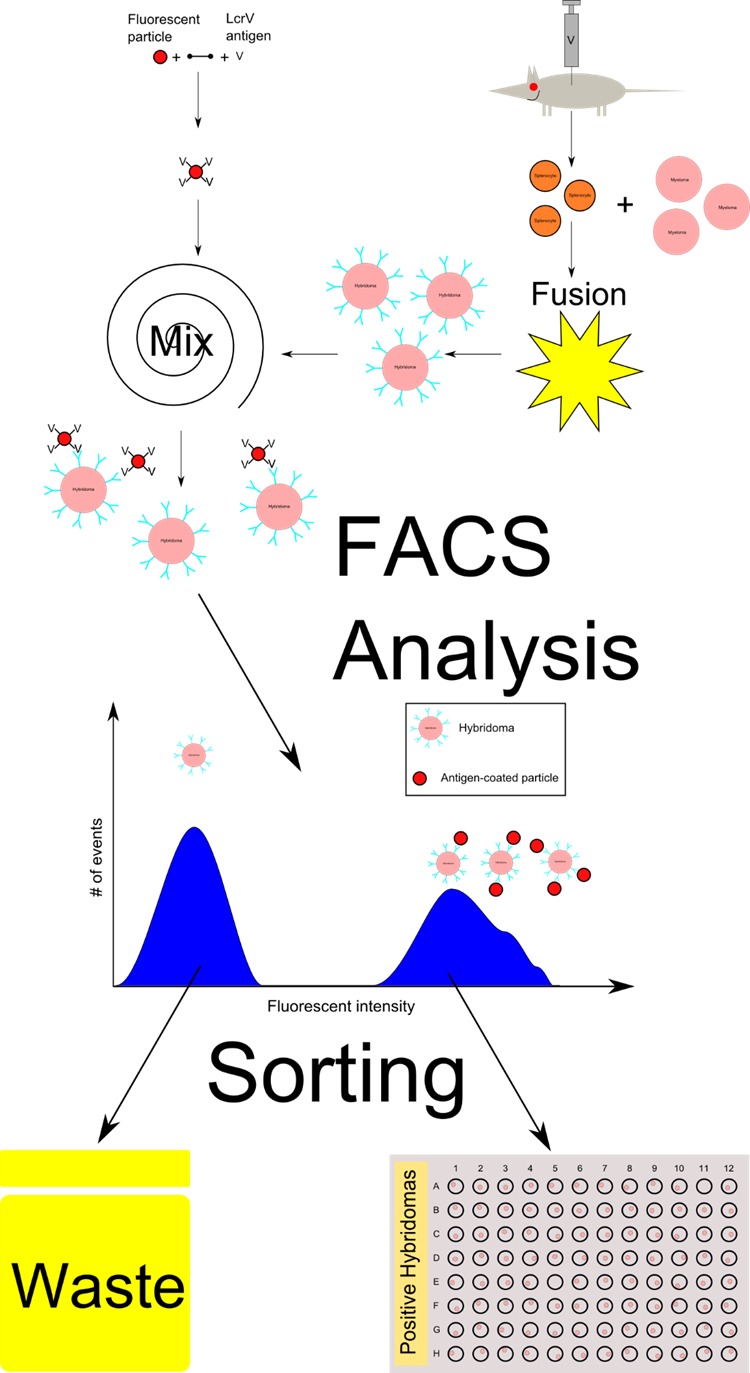
An illustration of the hybridoma sorting method (see Table 1). The sorting experiment was performed 2 times, every time using 10% of the spleen content, whereas the traditional hybridoma method used 90% of the spleen content. The efficiency is defined as the fraction of viable hybridomas which secrete antibodies with specificity towards the antigen (LcrV).

After sorting the hybridomas into 96-well plates, they were grown for one week, and the supernatant containing secreted anti-LcrV IgG was characterized using ELISA. In order to further validate the method, the resulting clones were grown and their medium was tested with ELISA against LcrV, and 85% and 93% were found positive in two independent experiments. In the control experiment, using the traditional protocol, only 7% of the clones were found positive, which is within the known range of traditional hybridoma success rate (**Table 1** and **Fig. 4**). Taking into account the sample fractions, per spleen, the sorting method should have yielded 140–170 positive clones, compared to only 22 positive clones using the traditional method (**Table 1**).

### Biolayer interferometry

**Figure 5.**
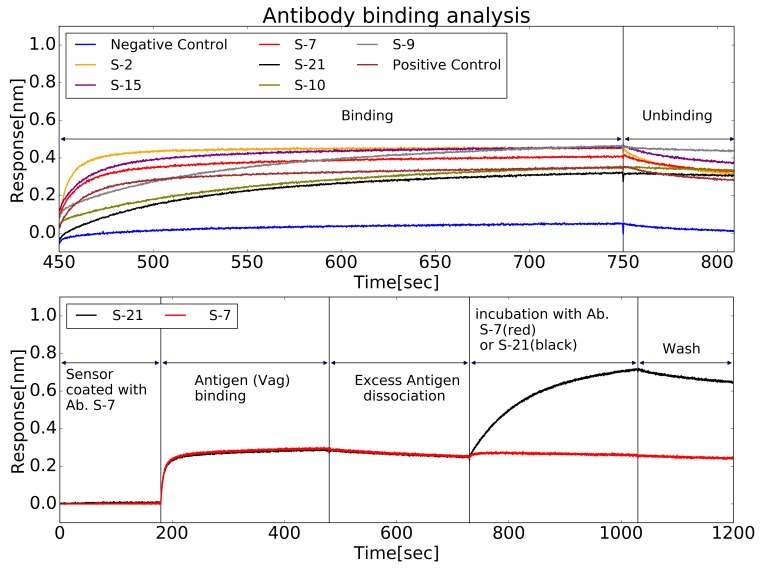
Screening of antibodies binding to LcrV. Real-time binding properties of antibodies to LcrV was measured using Octet BLI. Top: Biosensors coated with LcrV were used to estimate the Koff for a set of antibodies, from sorted clone media. Following a short wash, each sensor was immersed in a well containing concentrated supernatant of a different hybridoma clone (Binding step). This incubation was followed by a wash step, in which unbinding occurs, at a rate determined by the antibody’s Koff (Unbinding step). Bottom: Pairwise-mapping analysis for two antibodies against LcrV, chosen from the set of antibodies previously analyzed for Koff. Streptavidin-biosensors coated with biotinylated S-7 antibody were submerged in an LcrV-containing well and the wavelength interference was recorded (“Antigen Binding”). Following a short wash (“Excess Antigen Dissociation”), one sensor (red) was immersed with S-21 antibody while the other (black) was immersed with S-7 antibody (as a control). Both sensors were then washed. The rise in the signal for S-21 shows that S-21 can bind to S-7 coated LcrV and therefore the two antibodies do not compete on the same binding site.

Biolayer interferometry was used to test the clones from the sorted hybridomas. First, soluble anti-LcrV antibodies from six randomly selected cloned were tested for their dissociation rates (see **Fig. 5**, top). The measurements yielded a wide range of rates within the scale of 10^-4^ s or better. In order to demonstrate that chemical conjugation of LcrV to the particles can expose different epitopes, antibodies from two different clones were tested for mutual exclusiveness of binding epitopes. As can be seen in **Fig. 5** (bottom), the method can produce different antibodies that are not limited to a certain epitope. Two antibodies, S-7 and S-21 were purified using a standard IgG protocol [22]. The equilibrium dissociation constants (K_D_) for these antibodies were measured and found to be 0.23 and 2.4 nM, respectively. The affinities are comparable to the ones we measured for antibodies produced using the traditional method (see **Table S1**, in the Supplementary information).

## DISCUSSION

The work presented here shows how combining fluorescently-labeled antigens and FACS can dramatically improve hybridoma antibody production. This way, one can select specific hybridomas and even (as remains to be shown) for more advanced properties (isotype, specificity versus universality using multiple antigens, *etc*.). Our calculations show that even in absolute numbers, FACS-based hybridoma workflows can yield very large numbers of clones per spleen and therefore does not require a high survival rate for the sorted cells. Other hybridoma sorting methods use individual, fluorescently labeled, protein molecules, to label hybridomas. This way, the fluorescent signal is proportional to the level of antibody display on the cell surface, which is not necessarily related to the binding properties of the antibody which may lead to the loss of valuable clones, due to low or borderline signal. On the other hand, by using the fluorescent beads approach, even hybridomas with low membrane display of antibodies can be detected and sorted, since the binding of even one fluorescent particle to a hybridoma is enough to obtain a strong, significant signal. In such scenarios, it is important to quickly obtain and analyze large collections of antibodies against new variants or strains. Another, indirect, advantage is the reduction of animal numbers, as a result of the higher efficiency and yield. The particle sorting approach can be further generalized to the use of micron-scale particulate antigens (fluorescently labeled bacteria or spores) and to the use of magnetic particles, allowing more versatile sorting schemes. To summarize, this work demonstrates the great potential that particle based, hybridoma sorting holds for the rapid selection of antibodies against select agents and emerging pathogens (such as *Y*. *pestis*).

## Supplementary Material

Supplementary information **Figure S1.** Cytometric analysis for the binding of LcrV coated particles to anti-LcrV hybridomas.
**Figure S2.** A schematic description of the hybridoma sorting workflow.
**Table S1.** A comparison of dissociation constants between antibodies which were produced in the traditional hybridoma method and the sorting method.
Supplementary information of this article can be found online at
http://www.jbmethods.org/jbm/rt/suppFiles/257.

## Figures and Tables

**Figure 1.**
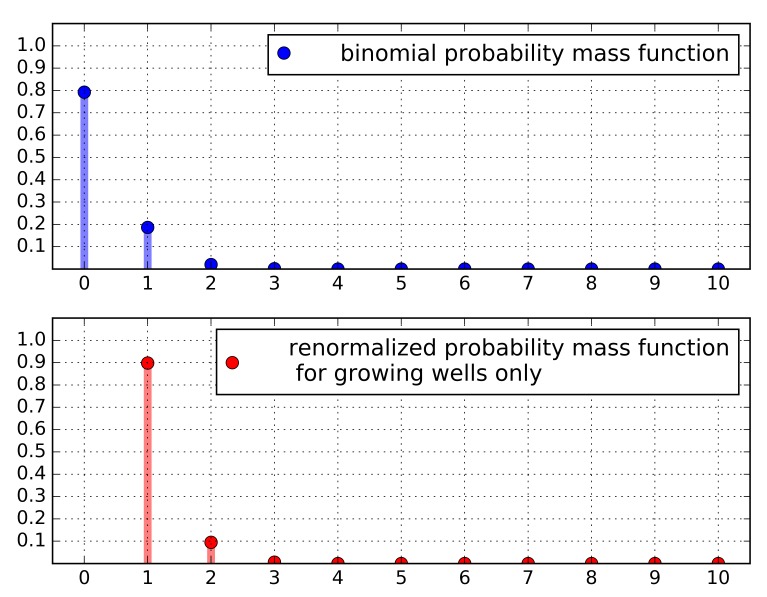
A simplistic model explaining how, by adjusting the number of cells sorted per well according to experimentally determined survival rates, “effectively monoclonal” sorting can be reached. Top: The binomial probability mass function, which approximately describes the number of viable cells (capable of growing and forming colonies) in a well. Bottom: When only “growing wells” (the ones in which colonies can be found) are taken into account, we can obtain a new probability mass function, defined for 0 < *n* ≤ 10. In this case, the probability of a “growing well” to be monoclonal is ~0.9 (or ~90%).

**Table 1.** The summary statistics for sorting.

	Sorting method	Traditional method
% of total fusion cells	10%	90%
Positive clones/total clones	13/14, 17/20 (93%, 85%)	20/300 (7%)
Calculated clones per entire spleen (estimated by extrapolation)	140–170	22
